# Vaccination status and factors associated among children age 12–23 months in Ethiopia, based on 2016 EDHS: Logit based multinomial logistic regression analysis

**DOI:** 10.1371/journal.pone.0264004

**Published:** 2022-02-25

**Authors:** Ermias Bekele Enyew, Abiyu Abadi Tareke

**Affiliations:** 1 Department of Health Informatics, Mettu University Ethiopia, Mettu, Ethiopia; 2 West Armachiho District Health Office, Gondar, Ethiopia; Oregon State University, UNITED STATES

## Abstract

**Background:**

Childhood immunization is one of the most cost-effective prevention measures for children’s mortality and morbidity, saving 2–3 million lives per year. In Ethiopia, under-five mortality rates, about 190,000 children die each year. Different research conducted in Ethiopia on childhood vaccination have focused on either vaccination coverage of individual vaccine or complete and incomplete vaccination. As far as my literature searching, studies separated the vaccination status into non-vaccinated, partially vaccinated and full vaccinated and assorted factors among children age 12–23 month in Ethiopia were limited. Therefore, the aim of this study was to identify factors associated with vaccination status among children 12–23 months of age in Ethiopia.

**Method:**

A secondary data analysis was done based on the 2016 Ethiopian Demographic and Health Survey (EDHS). A total weighted sample of 1911 children age 12–23 months of age were included in the study. Logit based Multinomial logistic regression analysis was computed to distinguish factors associated with routine vaccination of children aged 12–23 months. P-value less than 0.05 was used to declare statistical significance of each independent variables, and adjusted odd ratio (AOR) with 95% confidence interval were used to present the result and STATA 14 was utilized for data management and analysis.

**Result:**

Overall the prevalence of full vaccinated children was 35%, while 49% of children were partially vaccinated and 16% were non-vaccinated. In multinomial analysis, having focused ANC (at least four visits) contrasted to no ANC visits at all had 9.7 higher odd of being fully vaccinated than not vaccinated [AOR = 9.74, 95% CI = 3.52–26.94], and 5 times higher odd of being partially vaccinated than not vaccinated [AOR = 4.97, 95% CI = 2.00–12.33].

**Conclusion:**

The present study found that childhood full vaccination status was low compared with the World Health Organization targets. Frequency of ANC visit and visited by field worker were significantly associated both partially and full vaccination whereas, visited health facility last 12 months and wealth status were significantly associated with childhood full vaccination.

## Introduction

Childhood immunization is one of the most cost-effective prevention measures for children’s mortality and morbidity, saving 2–3 million lives per year [1]. Globally, over the last five years, the percentage of children receiving prescribed vaccines has remained constant. In 2019, approximately 85 percent (116 million) of infants received three doses of DTP-3/Penta-3 vaccine, and 125 countries achieved at least 90% DTP3 vaccination coverage, but approximately 19.7 million children under the age of one year have not received basic vaccines [2].

In low- and middle-income nations, childhood vaccination provides good protection against vaccine-preventable diseases [3, 4]. While countries in Sub-Saharan Africa give high attention to childhood vaccination, child mortality related with vaccine-preventable diseases remains high and hard to reach (remote areas) of developing countries, only 50% of children and one in twenty had access to childhood vaccination [5].

In Ethiopian, EPI program was launched in 1980 with a longer-term goal of achieving 90% DPT3 coverage in all regions through strategies of reaching every district (RED) and sustainable outreach service (SOS) approaches and protect nearly 3-million annual births against VPDs [6]. However, a large number of children have not been immunized [7]. The vaccination status of children is used to track the performance of vaccination services at the local, national, and international levels in order to develop vaccine-preventable disease eradication, elimination, and control strategies [8, 9].

According to the 2016 EDHS report, childhood vaccination was low in the emerging regions of Ethiopia [10]. And under-five mortality rates, about 190,000 children die each year [11]. Studies have shown that maternal education, socioeconomic status, antenatal care, place of delivery, sex of child, postnatal care, media exposure, perceptions of vaccination, place of residence and cold chain management also influence childhood vaccination status [7, 12–15].

Different research conducted in Ethiopia on childhood vaccination have focused on either vaccination coverage of individual vaccine or complete and incomplete, vaccination and associated factor using two categories full vaccination or non—vaccination. Even if the previous studies give important clues to policymaking and other stockholder, they mainly lack of consistency and representative to be used for national level policymaking and programming by concerned bodies, as far as my literature searching, studies separated the vaccination status into non-vaccinated, partially vaccinated and full vaccinated and assorted factors among children age 12–23 month in Ethiopia are limited.

Using logit based multinomial logistic regression analysis assessing the factors that contribute to vaccination status is important in order to devise evidence-based strategies and polices that would raise the overall immunization coverage which in turn reduce the infant and child mortality. This findings may help us to plan highly focused and specific interventional initiatives based on factors predicting vaccination status of children separately i.e. for partially vaccinated, for fully vaccinated and not vaccinated. Therefore, the aim of this study was to identify factors associated with vaccination status among children 12–23 months of age in Ethiopia by using logit based multinomial logistic regression analysis based on the 2016 Ethiopian Demographic and Health Survey (EDHS).

## Method and material

### Study design and sampling procedures

The study was conducted in Ethiopia. Ethiopia is located in the Horn of Africa. The current population of Ethiopia is 116,870,377 in 2021, with 78.3% living in rural areas based on World meter elaboration of the latest United Nations data. It has nine Regional states (A far, Amhara, Benishangul-Gumuz, Gambella, Harari, Oromia, Somali, Southern Nations, Nationalities, and People’s Region (SNNP) and Tigray) and two city Administrative (Addis Ababa and Dire-Dawa). Ethiopia has followed 3 tiers of preventive healthcare system approaches. These are primary-level healthcare comprising of a primary hospital, health center, and health post; secondary-level healthcare (general hospital); and tertiary-level healthcare (specialized hospital).

A secondary data analysis was done based on the 2016 Ethiopian Demographic and Health Survey (EDHS). In 2016 EDHS, a two stage stratified sampling technique was employed to select representative samples for the country as whole. The regions in the country were stratified into urban and rural areas. Then, samples of enumeration areas (EAs) were selected in each stratum in two stages. In the first stage, 645 EAs were selected with probability proportional to the EA size. The EA size is the number of residential households in the EA as determined in the 2007 Ethiopian Population and Housing Census. In the second stage, a fixed number of 28 households per cluster were selected randomly from the household listing [10]. All women aged 15–49 years who were usual members of the selected households were eligible for female survey. Out of 7,193 women who gave birth in the past 5 years preceding of the survey, 5,980 were interviewed about the vaccination status of their children and children of age 12–23 months with missing age of child and outcome variable were excluded from the study. A total weighted sample of 1911 children age 12–23 months of age were included in the study.

### Study variables

#### Outcome variables

*Vaccination status*. Number of children aged 12–23 months received one dose of BCG vaccine, three doses of polio vaccine, and three doses of pentavalent vaccine (DTP-hepB-Hib), three dose of pneumococcal conjugate vaccine (PCV), two dose of virus vaccine and one dose of measles vaccine was considered as “fully vaccinated”; partially vaccinated status was defined as having received some but not all vaccines; and non-vaccinated status was defined as not having received any vaccines. Fully vaccination definition is adopted from Ethiopian national HMIS (health management information system) indicators guideline [16]. But, the vaccine IPV (injectable polio vaccine) was not collected in 2016 EDHS and not included in this analysis.

#### Independent variables

The independent variables included in this study were: maternal age, women educational status, husband/partner’s education status, place of residence, women working status, wealth status, media exposure, frequency of ANC visit, region, and visited health facility last 12 months, visited by field worker, child sex, and place of delivery and participation of decision-making.

#### Operational definition

*Participation on decision making*. women who decided their health issue alone are labeled as”yes” and coded as “1”, who decide jointly with their partner were labeled as “some” and coded as “2”, while those responded their partner alone decide on their health issue were labeled as “no” and coded as “0”.

### Statistical analysis

Logit based Multinomial (polytomous) logistic regression analysis was computed to distinguish factors associated with routine vaccination of children aged 12–23 months. Multinomial Logistic Regression is a simple continuation of binomial logistic regression model to be used when the dependent variable is nominal and has more than two categories. Children who were not vaccinated to any routine vaccination were considered as referent category and specified as “0” and the remaining categories i.e. fully vaccinated and partially vaccinated were considered as alternative category (not the reference category). Variance inflation factors (VIF) were assessed to check multicollinearity among the variables. A VIF value greater than 10 was considered as an indication of multi-collinearity; however, no significant multicollinearity was observed. P-value less than 0.05 was used to declare statistical significance of each independent variables, and adjusted Odd ratio (AOR) with 95% confidence interval were used to present the result. The parameter AOR indicates the likely to membership of one category of the independent variable compared reference category. The closer a value of AOR to zero the less effect of the explanatory variable has on the dependent variable’s alternative category as compared to reference category [17].

### Ethical approval and consent to participate

Authors have requested DHS Program through an online request by written letter of objective and significance of the study. Permission for data access was granted to download and use the data from http://www.dhsprogram.com. The DHS programs permitted data access, and data were used for only the current study.

## Result

A total of 1,911 (weighted) children aged 12 to 23 months were included in this study. The majority of the children (11.1%) are at the age of one year. The children’s and mother’s mean age were 16.9 months and 29.18 years, respectively.

Generally, in Ethiopia prevalence of full vaccinated children was 35%, while 49% of children were partially vaccinated and 16% were non-vaccinated. In terms of maternal age fully vaccinated children were exhibited among women aged 15–19 (40.6%) and 20–29 (38.4%) years. Conversely, partially vaccinated children are highly noticed among women aged 40–49 (56.4%) & 30–39 (49.3%) years. Regarding educational status of maternity of uneducated women 624(52.5%) and 329 (28%) their children belongs to partially vaccinated and fully vaccinated respectively. Of 713 women who give birth at health facility to their last birth, more than half of their children were partially vaccinated. (Table 1).

**Table 1:** Distribution of vaccination status of children aged 12–23 months in Ethiopia by selected characteristics, EDHS 2016.

Characteristics	Vaccination status			total
	Not vaccinated	Partially vaccinated	Fully vaccinated	
Vaccination status	309 (16%)	936 (49%)	666(35%)	1911
**Maternal age**				
15–19	19(25.8%)	27(33.7%)	31(40.6%)	77
20–29	124(13.1%)	464(48.5%)	366(38.4%)	954
30–39	146(20.1%)	360(49.3%)	224(30.6%)	730
40–49	18(12.2%)	85(56.4%)	47(31.4%)	150
**Child sex**				
Male	143(16.5%)	437(49.8%)	293(33.7%)	873
Female	166(16.0%)	499(48.0%)	374(36.0%)	1038
**Maternal educational status**				
no education	232(19.7%)	624(52.5%)	329(27.8%)	1185
Primary	66(11.8%)	265(47.4%)	227(40.8%)	558
Secondary	7(6.6%)	32(32.3%)	63(61.2%)	102
Higher	4(5.5%)	14(21.2%)	48(73.4%)	66
**Occupation**				
Working	110(12.7%)	413(47.2%)	349(40.1%)	873
Not working	198(19.2%)	522(50.2%)	318(30.6%)	1038
**Place of delivery**				
Home	233(19.5%)	655(54.5%)	310(26.0%)	1198
Health facility	76(10.7%)	280(39.3%)	357(50.0%)	713
**Frequency of ANC visit**				
No ANC	188(28.5%)	360(54.4%)	113(17.1%)	665
1_3 visit	65(12.1%)	265(49.4%)	207(38.6%)	537
>= 4 visits	28(4.6%)	260(42.7%)	320(52.6%)	611
**Health facility visit in the past 12 months**				
No	197 (20.3%)	538(55.5%)	234(24.2%)	968
Yes	112(12.0%)	398(42.0%)	432(46.0%)	942
**Visited by field worker in the last 12 months**				
Not visited	255(13.3%)	671(35.1%)	388(20.3%)	1314
Visited	54(2.8%)	264(13.8%)	596(14.5%)	596
**Wealth status**				
Poorest	120(24.9%)	264(54.8%)	97(20.3%)	481
Poorer	66(17.8%)	185(49.1%)	123(33.1%)	373
Middle	60(14.2%)	227(53.9%)	134(31.9%)	421
Richer	39(11.2%)	169(47.6%)	145(41.2%)	353
Richest	24(8.6%)	91(32.3%)	167(59.2%)	282
**Place of residency**				
Rural	300(17.9%)	850(50.7%)	525(31.4%)	1674
Urban	9(3.8%)	80(34.9%)	140(61.3%)	228
**Region**				
Urban admin	1(1.6%)	14(21.4%)	51(77.1%)	66
Agrarian	283(16.5%)	857(49.5%)	585(34.0%)	1725
Pastoralist	65(20.3%)	65(54.0%)	30(25.7%)	119
**Media exposure**				
No	245(12.7%)	665(47.2%)	349(40.1%)	1260
Yes	63(19.2%)	270(50.2%)	316(30.6%)	650
**Participation on decision making**				
No	66(18.4%)	194(54.1%)	98(27.5%)	358
Some	198(16.4%)	563(46.3%)	451(37.3%)	1212
Yes	25(10.7%)	123(51.5%)	89(37.8%)	237
**Husband/partner’s educational status**				
None	165(19.6%)	441(52.3%)	236(28.1%)	842
Primary	98(13.7%)	353(49.1%)	268(37.2%)	718
Secondary	16(11.5%)	52(36.2%)	75(52.3%)	1438
Higher	9(9.1%)	29(29.9%)	59(61.0%)	96

Agrarian; Tigray, Amhara, Oromo, and South nation nationality and peoples. Pastoralist; Afar, Gambella, Benishangul, and Somalia and urban administration; Addis-Ababa, Harari and Dire-Dawa

**Fig 1:**
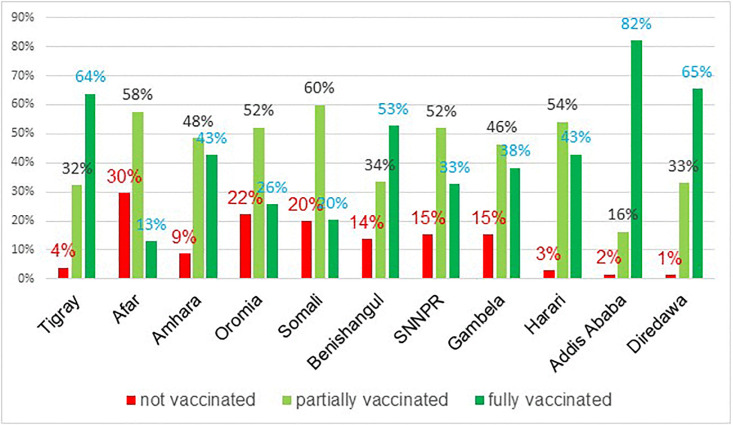
Prevalence of vaccination status among 12–23 months aged children by administrative region in Ethiopia, 2016 EDHS.

Highest fully vaccinated children was observed in Addis Ababa, Dire Dawa, Tigray and Benishangul regions, while lowest prevalence were noticed in Somalia, Afar and Oromia region (Fig 1).

### Factors associated with vaccination status among children age 12–23 months in Ethiopia, EDHIS 2016

Output of Logit based logistic regression analysis shown in (Table 2) using the category not vaccinated as base outcome (reference group) adjusted Odd Ratio (AOR) was estimated for child being partially vaccinated along with not vaccinated and fully vaccinated along with not vaccinated. The variables associated with partially and fully vaccination were; ANC visits, visited by field worker in the last 12 months, visit to health facility in the last 12 months, place of residency and wealth status. Being visited by field workers and ANC visits were variables that predicts both fully and partially vaccination status uniformly. But, the variable place of residency predicts only partially vaccinated, while visits to HF within the last 12 months and wealth status predicts only fully vaccination.

**Table 2:** Multinomial logistic regression table showing factors associated with vaccination status among children age 12–23 months in Ethiopia EDHIS 2016, (reference group = not vaccinated).

Characteristics	Partially vaccinated	full vaccinated
	Adjusted Odd Ratio with 95% CI	Adjusted Odd Ratio with 95% CI
**Maternal age**		
15–19	1.00	1.00
20–29	1.28 [0.36, 4.52]	0.75 [0.23,2.47]
30–39	0.96 [0.26, 3.62]	0.48 [0.13,1.85]
40–49	1.56 [0.32, 7.56]	0.82 [0.17,4.03]
**Sex of child**		
Male	1.00	1.00
Female	0.94 [0.60,1.47]	1.05 [0.63,1.75]
**Working status**		
Working	1.00	1.00
Not working	0.84 [0.50,1.40]	0.77 [0.46,1.31]
**Place of delivery**		
Home	1.00	1.00
Facility	0.61 [0.30, 1.25]	0.83 [0.39,1.76]
**ANC visits**		
None	1.00	1.00
1 to 3 visits	2.25 [1.25, 4.05]**	3.94 [2.05.7.56]***
≥ 4 visits	4.97 [2.00, 12.33]**	9.74 [3.52, 26.94]***
**Visit to HF**		
No	1.00	1.00
Yes	1.18[0.69,2.01]	2.16[1.32, 3.52]**
**Place of residency**		
Rural	1.00	1.00
Urban	3.82 [1.32,11.12]*	2.87 [0.81,10.20]
**Region**		
urban admin	1.00	1.00
Agrarians	0.38 [0.07,2.09]	0.19 [0.03,1.11]
Pastoralists	0.36 [0.06,2.11]	0.19 [0.03, 1.17]
**Visited by field worker**		
No	1.00	1.00
Visited	2.13 [1.13,4.02]*	2.97 [1.58,5.60]**
**media exposure**		
No	1.00	1.00
Yes	1.05 [0.51,2.18]	1.35 [0.69,2.64]
**Decision making autonomy**		
No	1.00	1.00
Some	0.61 [0.34,1.10]	0.83 [0.41,1.71]
Yes	1.17 [0.53,2.56]	1.32 [0.53,3.29]
**Husband’s/partner’s education**		
None	1.00	1.00
Primary	1.20 [0.66,2.19]	1.21 [0.66, 2.20]
Secondary	0.67 [0.22,2.04]	0.65 [0.19,2.18]
Higher	0.94 [0.15, 5.93]	0.52 [0.09,3.11]
**Maternal education**		
None	1.00	1.00
Primary	1.64 [0.77,3.47]	1.77 [0.82,3.80]
Secondary	0.65 [0.13,3.18]	1.13 [0.25,5.05]
Higher	0.23 [0.02,2.17]	0.76 [0.09,6.46]
**Wealth status**		
Poorest	1.00	1.00
Poorer	1.30 [0.63,2.66]	1.89 [0.88,4.03]
Middle	1.97 [0.91,4.26]	2.31 [1.07,5.01]*
Richer	1.63 [0.80,3.32]	2.59 [1.22,5.49]*
Richest	0.74 [0.24,2.25]	1.69 [0.50,5.79]
Constants	4.06 [0.40,41.17]	1.86 [0.19,18.53]

p-value*<0.05, **<0.01, *** = <0.001 and HF; health facility

In contrast to children from rural residents, children from urban area had 3.8 times higher odd of being partially vaccinated compared to not vaccinated children [AOR = 3.82, 95% CL: 1.32–11.12]. In the same way, Children whose mothers visited a health facility in the past 12 months in comparison to those who did not have any visit had 2.16 greater odd of being fully vaccinated relative to non-vaccinated children (AOR = 2.16, 95% CI = 1.4–3.52].

Children belongs to middle wealth households relative to poorest had 2.3 higher odd of being fully vaccinated in contrast to not vaccinated children [AOR = 2.31, 95% CI = 1.07–5.01]. In the same manner, children from richer households relative to poorest had 2.6 higher odd of being fully vaccinated than not vaccinated. [AOR = 2.59, 95% CI = 1.22–5.49].

Children whose mothers made 1–3 ANC visits compared to no ANC visit had nearly four times higher add of being fully vaccinated compared to non-vaccinated [AOR = 3.94, 95% CI = 2.05–7.56], and 2.2 folds higher odd of being partially vaccinated compared to non-vaccinated [AOR = 2.25, 95% CI = 1.25–4.05] in similar fashion, having focused ANC (at least four visits) contrasted to no ANC visits at all had 9.7 higher odd of being fully vaccinated than not vaccinated [AOR = 9.74, 95% CI = 3.52–26.94], and 5 times higher odd of being partially vaccinated than not vaccinated [AOR = 4.97, 95% CI = 2.00–12.33]. The odd of being fully vaccinated compared to not vaccinated is threefold higher in children whose mother was visited by field workers in the past 12 months compared to not visited [AOR = 2.97, 95% CI = 1.58–5.60], and 2.13 fold higher odd of being partially vaccinated in contrary to not vaccinated [AOR = 2.13, 95% CI = 1.13–4.02] holding other covariates constant.

## Discussion

We assessed the routine vaccination coverage and risk factors for vaccination status among children aged 12–23 months in the Ethiopia using evidence from fourth Ethiopian demographic health survey. To declare as child fully vaccinated the context of Ethiopia was applied in this study. Infanthood vaccination has its own contribution to public health like reducing vaccine preventable diseases. Among the vaccine preventable diseases that can be minimized by routine vaccination; Diphtheria, hepatitis B, influenza, pertussis, pneumonia, tetanus, rotavirus, tuberculosis and measles [18].

This study examined predicting factors of vaccination status of children age 12–23 months using Logit based multinomial logistic regression analysis. Factors affecting vaccination status was identified separated for partial vaccination and full vaccination status taking not vaccinated children as reference category. From Table 1 nearly half (49%) of the children were partially vaccinated, more than two-third (33%) were fully vaccinated and 16% were non-vaccinated. Among predictor variables identified from this study being visited by field workers and ANC visits were common predictor variables to both alternative categories. But, visit to health facility in the past 12 months, place of residency and wealth status were not consistent (common) predictors of alternative categories.

The current study found that children born from mothers who had at least four ANC visits are 10 folds more likely to be fully vaccinated than not vaccinated compared to no ANC visits, and had five times higher odd of partially vaccinated than not vaccinated children compared to who had no visits. This finding is consistent with research done in Ethiopia [19], Bangladesh [20], Afghanistan [21] and Senegal [14]. The same pattern of association was observed among women who had one to three antenatal care visits. A possible explanation for these results may be due to; women during the time of ANC visits they have greater chance of communicating with their healthcare providers about the importance of childhood vaccination. It is better to make additional effort to increase antenatal care visits by Policy makers, health educators and other public health concerned body.

Another crucial finding from this study was visits by fieldworker, children from visited households by a fieldworker in the past 12 months are 2.13 higher odd of partially vaccinated than not vaccinated as compared to not visited groups, and 3 times more odd of fully vaccinated than not vaccinated in contrary to children from households not visited by field worker in the past 12 months. Similarly, women who had visited to health facility in the past 12 months are more likely to get their children fully vaccinated than not vaccinated children in comparison to children whose mother hadn’t visited. This finding broadly supported by the work of other studies in this area [20]. There are several possible explanations for this result; mothers who had visited by fieldworkers are more likely to get health information related to vaccination and to discuss about vaccination status of under five children in that specific visited household. Unvaccinated/partially vaccinated children are more likely to be linked to nearby health facilities either to complete or to start up their routine vaccination schedule by the fieldworker.

Regarding place of residency compared to children inhabiting in rural areas, urban inhabitant children 3.8 times higher odd of partially vaccinated than not vaccinated to routine immunizations. Women from urban area are more likely to health information and good awareness about health of their children in general and vaccination status of their children in specific. Additionally, urban dweller women are more likely to get vaccinated their children due to availability and accessibility of health care system in contrast to rural dweller mothers. There is documented evidence about urban-rural difference in utilization of maternal health services in prior EDHS studies [22].

Finally, wealth status of the household had significant influence on vaccination status of children. Children from middle and richer household were more likely to be fully vaccinated. From this study, as the level of wealth increases the odd of being fully vaccinated than not vaccinated increases accordingly. However, being children to the category of richest household was not statistical significant, this may be due to small observations. These results seems in line with previous studies [21, 23]. These relationships may partly be explained by; Women in higher territory of wealth are likely to empowered and less likely to confront challenge of transportation fee to take their child to vaccination site.

Even though, this study used national representative dataset and advanced statistical model to predict factors associated with vaccination status of children it is not free from limitations. The main limitation of this study is; unable to include predictor variables that are not collected by EDHS like knowledge, attitude toward vaccination, maternal TT vaccination. And due to cross-sectional nature of study design it is difficult to build causation types of relationships.

## Conclusion

The present study found that childhood full vaccination status was low compared with the World Health Organization targets. After controlling for potential confounder, the logit based multinomial logistic regression result showed that frequency of ANC visit and visited by field worker were significantly associated both partially and full vaccination whereas, visited health facility last 12 months and wealth status were significantly associated with childhood full vaccination. As a result, enhancing ANC visit, visited by field worker and visited health facility last 12 months can help to improve childhood vaccination.

## Abbreviations

ANC: Antenatal care
AOR: Adjusted Odd Ratio
CI: confidence Interval
DPT: Diphtheria, Pertussis and Tetanus
EA: Enumeration Area
EDHS: Ethiopia Demographic and Health Survey
EPI: Expanded program of immunization
